# The Novel Use of Umbilical Cord Blood to Obtain Complete Blood Counts for Critical Neonatal Assessment

**DOI:** 10.7759/cureus.28009

**Published:** 2022-08-14

**Authors:** Alexandra P Hansen, Gayle D Haischer-Rollo, Jonathan B Shapiro, James K Aden, Jude M Abadie, Thornton S Mu

**Affiliations:** 1 Neonatal-Perinatal Medicine, Brooke Army Medical Center, Joint Base San Antonio (JBSA) - Fort Sam Houston, USA; 2 Statistics, Brooke Army Medical Center, Joint Base San Antonio (JBSA) - Fort Sam Houston, USA; 3 Clinical Pathology, Texas Tech University Health Sciences Center El Paso Paul L. Foster School of Medicine, El Paso, USA; 4 Pediatrics, Uniformed Services University of the Health Sciences, Bethesda, USA

**Keywords:** anemia, high-risk neonates, cbc testing, cbc, neonatal intensive care unit (nicu), clinical laboratory, sepsis screen, iron deficiency anemia (ida), cord blood

## Abstract

Background: Neonates undergoing clinical evaluations are often subjected to potentially painful phlebotomy for laboratory tests. The use of cord blood laboratory values for admission has been suggested as a means to decrease the risk of painful venipuncture and anemia.

Methods: Peripheral and umbilical cord blood complete blood count (CBC) results were obtained from infants who required a CBC. Results were compared using the Sysmex XN heme analyzer (Sysmex, Kobe, Japan).

Results: White blood cell (WBC) and hemoglobin (HgB) values were significantly higher in peripheral samples than in cord samples. The mean cord WBC count was 14.1 × 10^3^/mm^3^ versus 15.6 × 10^3^/mm^3^ peripherally (p < 0.001). The mean cord HgB was 15.8 g/dL versus 16.8 g/dL peripherally (p < 0.001). Cord platelet (Plt) counts were, conversely, lower in peripheral samples than in cord samples (264.8 × 10^3^/mm^3^ versus 242.3 × 10^3^/mm^3^, respectively; p < 0.001). Although statistically different, the mean CBC values from both samples were within the reference ranges. Delayed cord clamping (DCC) increased peripheral versus cord HgB difference nearly threefold (0.6-1.7 g/dL; p = 0.01).

Conclusions: Cord blood is an acceptable source for CBC blood sampling in newborn infants and can be used for clinical decisions. CBC laboratory values for cord blood remained within the peripheral blood reference range, with slight variability between the two samples.

## Introduction

Newborn infants undergoing sepsis evaluations or admission to the neonatal intensive care unit (NICU) often require phlebotomy procedures [1-3].Admission tests may include complete blood count (CBC) with manual differential (MD), blood culture, blood gas, blood type and screen, blood glucose, and inflammatory markers such as C-reactive protein and procalcitonin [4,5]. The total fetoplacental blood volume is estimated to be between 105 and 162 mL/kg, and the neonatal blood volume is estimated to be 66-95 mL/kg; therefore, blood required for admission tests can represent 2%-3% of a term infant’s blood volume and up to 5%-10% in premature infants [6]. Delayed cord clamping (DCC) can increase neonatal blood volume by 15%-30% [7].With up to 10% of infants undergoing screening due to perinatal risk factors, decreasing phlebotomy could have a far-reaching impact in decreasing both anemia and discomfort [8]. In critically ill preterm neonates, initial and subsequent phlebotomy procedures increase the likelihood of anemia and the need for packed red blood cell (pRBC) transfusions [9]. Strategies to decrease the risk of anemia and the need for transfusions include DCC, cord stripping, erythropoiesis-stimulating agents, limiting phlebotomy via the use of point-of-care testing devices, and transcutaneous measurements [10,11].Phlebotomy can equal up to 20 mL/kg of blood per week during peak illness; therefore, decreasing phlebotomy can reduce iatrogenic anemia seen commonly in NICUs [12].Testing umbilical cord blood drawn during post-placental delivery could be one strategy to decrease phlebotomy and the risk of anemia [13,14]. Studies suggest that cord blood is equivalent to peripherally drawn blood for CBC parameters and blood culture analyses [8,10]. Published normative values for term cord blood CBC samples are distinct from peripheral samples during the first three days of life; however, differences represent a variation that is predictable and reproducible [7,15,16].

The majority of infants requiring laboratory evaluations do not have easy access via umbilical lines and require phlebotomy by direct venous/arterial puncture or heel sticks [17]. These procedures are presumed to be painful for infants. While some studies have investigated the utility of topical anesthetics to reduce pain associated with venipuncture, a more favorable option would be to reduce the number of venipunctures and thus avoid the negative effects of pain [18].

Cord blood sampling had not previously been used for CBCs at our institution despite growing support in the literature. The primary outcome of this study was to demonstrate that cord blood is an acceptable source for initial CBC evaluation in newborn infants by comparing cord and peripheral CBC results. These evaluations are viewed in the context of recent clinical practice changes that include DCC following birth.

This article was previously presented as a poster at the 2019 American Academy of Pediatrics (AAP) National Conference and Exhibition (NCE) Section on Neonatal-Perinatal Medicine (SONPM) Poster Session on October 25, 2019.

## Materials and methods

The study population included any newborn infant prenatally screened to require admission CBC testing for clinical purposes such as prematurity (less than 35 weeks gestation), maternal chorioamnionitis, or early-onset sepsis screen. Participants were recruited from Brooke Army Medical Center (BAMC), a level 1 trauma tertiary care center with a level 3 NICU during two time periods, from October 2017 to August 2018 and from January 2019 to June 2019. Approximately 1,800 infants are born annually with 300 annual NICU admissions. Chorioamnionitis is determined by placental pathology or based upon a clinical determination from the obstetrics team. Perinatal risk factors for newborn sepsis can be considered by maternal group B streptococcal (GBS) colonization, prolonged rupture of membranes > 18 hours, maternal temperature (≥99.5°F), uterine/fundal tenderness, persistent fetal tachycardia > 160 beats/minute, or foul-smelling vaginal drainage/purulent discharge [3]. We obtain both CBC and blood cultures on infants born to a mother diagnosed with chorioamnionitis as part of our newborn care policy. Additionally, we instituted our DCC protocol just prior to the beginning of this project. For vigorous infants with no contraindications, such as urgent resuscitation requirement, intrauterine growth retardation, placental anomalies/abruption, monochorionic twin gestation, maternal/sibling history of Rhesus factor (Rh) hemolytic disease, and maternal human immunodeficiency virus (HIV)/hepatitis B/C infections, DCC is routinely performed for 60 seconds.

The study protocol was approved by our Institutional Review Board meeting minimal risk standards. Verbal consent for the use of cord blood samples was obtained as cord CBC results were de-identified and used for research purposes alone. The demographic data collected from the inpatient record included maternal age, race, ethnicity, and medical history, as well as neonatal growth parameters, birth history, and admission diagnoses. All pregnant women presenting to obstetrics service for delivery and whose infants required a CBC at the time of birth were verbally consented to enroll their neonates into the study. The only exclusion criteria were infants whose parents had elected to bank cord blood for storage.

The hospital core laboratory’s Sysmex XN heme analytical platform (Sysmex, Kobe, JP) supported the hematological evaluation of both cord and peripherally drawn CBC samples [19]. During sample analysis, nucleated red blood cells (NRBCs) are identified and counted to allow for an accurate count of white blood cells (WBCs) in the presence of NRBCs. The methodology uses fluorescent flow cytometry with polymethine dye for nucleic acid and a cell-specific lyse compound to disrupt cellular membranes to allow for intracellular measurements. Fluorocell® is the reagent used to label nucleated cells within the lysed blood samples and subsequently allow the determination of NRBC, WBC, and basophil counts. For platelet (Plt) measurements, a sample volume of the whole blood specimen is introduced into the analyzer and diluted into a 1:200 CELLPACK® diluent. Fluorocell® platelet reagent is then introduced into the sample, and the entire dilution is maintained at a constant temperature so that the label can be incorporated into the platelets present in the sample. The labeled sample is then introduced into the sheath flow detector where forward scattered light and side fluorescence are measured, allowing the platelet count to be determined. The flow cytometry methodology is similar to WBC measurements. A sample volume of the whole blood is diluted at 1:60 and lysed by adding the Sysmex lysing reagent Lysercell®. The Fluorocell® reagent is then added, and the entire dilution is maintained at a constant temperature in order to label the nucleated cells in the sample. The labeled sample is then introduced into the sheath flow detector where forward scattered light and side fluorescence are measured, allowing the WBC count, NRBCs, and basophil count to be determined.

Cord blood was drawn from the umbilical vein or artery by needle aspiration immediately following placental delivery to perform any clinically indicated tests, such as umbilical cord blood gases and blood type. The cord segment was carefully punctured with an 18G needle, bevel up, and attached to a 5-mL syringe to enter the umbilical vein or artery. Care was made to ensure that the needle remained visible under the surface of the cord to avoid any Wharton’s jelly. Gentle upward traction was applied to maintain vessel patency during aspiration. Purple top vacutainers were filled with a minimum of 1-2 mL of whole blood and delivered to the laboratory along with admission laboratory results drawn from the neonate. For samples that were also used for culture, the area of the cord was first painted with povidone-iodine and allowed to dry for at least 60 seconds prior to sampling. Blood culture bottles were filled with 1-2 mL of whole blood.

Power analyses were performed by an institutional statistician. Per the Clinical Laboratory Improvement Act (CLIA) regulations [20], to achieve 80% power to detect the mean difference in hemoglobin (HgB) concentration of 0.8 g/dL, 1K cells/mm^3^ WBC, and 40K cells/mm^3^ Plt count with a significance of 5% using paired t-test method, a total of 93 infants were determined adequate for validation and analysis. All laboratory values were summarized using means and standard deviations (SD) or standard error of the means (SEM). The cord and peripheral blood samples were compared using paired t-tests with Bland-Altman analysis or Wilcoxon’s signed-rank test. Furthermore, t-tests were used to compare delta changes among selected subgroups from the cord and peripheral blood samples. All p-values less than 0.05 were considered statistically significant. All analyses were performed using JMP version 13.2 (SAS Corp., Cary, NC, USA).

## Results

A total of 156 infants met the inclusion criteria as shown in Table 1. Fifty-eight infants were excluded due to clotted specimens or difficulties obtaining cord blood. With the timely transfer of blood into the vacutainers within 1-2 minutes of collection, the 18% clot rate within the first four months of the study decreased to 8% for the remaining study period. Five samples had a prolonged time lapse between cord and peripheral CBC collection (4-20 hours), which were included in the analysis after review as the gap did not create outliers in the data.

**Figure 1:**
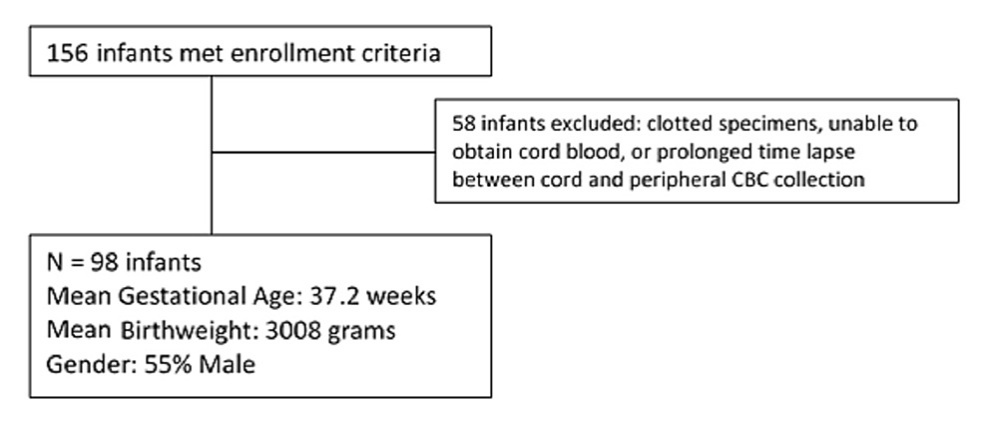
Infant Enrollment

The average maternal age was 29 years. Self-reported maternal ethnicities were 33% Hispanic and 67% non-Hispanic, similar to reported infant ethnicities. The mean gestational age (GA) and birth weight (BW) were 37.2 weeks and 3,008 g. Table 1 presents the full maternal and infant demographics. The mean time between cord and peripheral CBC collection was 1.7 hours. The most commonly documented indications for screening laboratory tests were sepsis evaluation (prenatal/perinatal chorioamnionitis) (80%) and prematurity (39%), with a number of infants having more than one indication. Of the 94 infants with documentation regarding the procedure, 40.8% (n = 40) underwent DCC.

**Table 1:** Maternal and Infant Demographics IQR: interquartile range; GA: gestational age; DCC: delayed cord clamping; DM: diabetes mellitus; PCOS: polycystic ovary syndrome

Characteristics	N = 98
Maternal age (years) (IQR)	29 (24-33)
Ethnicity (N)	
Hispanic	32
Non-Hispanic	66
Race (N)	
White	76
Black	8
Native Hawaiian or Pacific Islander	5
Asian	5
Mixed race	3
Other	1
Birth weight (grams) (IQR)	3,008 (2,510-3,636)
GA (weeks) (IQR)	37.2 (34.9-39.9)
DCC	40.8%
Pregnancy comorbidities (N)	
Depression/anxiety	15
Obesity, DM, PCOS	5
Hypertension	2
Migraine	5
Asthma	4
Autoimmune disorder	2
Hypothyroidism	3
Tobacco use	3
Leukemia	1
Admission diagnosis	
Sepsis screen	80%
Prematurity	39%
Respiratory distress syndrome	28%

Differences between cord and peripheral blood samples are shown in Table 2. Peripherally obtained blood sampling was drawn from arterial (72%), venous (7%), capillary (8%), and not documented (12%) samples. Peripheral HgB was higher compared to cord samples. The average difference was 1 g/dL (15.8 g/dL (cord) versus 16.8 g/dL (peripheral)). The ranges of HgB values from cord versus peripheral samples were 11.5-20.5 g/dL and 12.7-22.5 g/dL, respectively. Similarly, hematocrit (Hct) percentages were 1.3% higher in peripheral samples. The ranges of Hct values from cord versus peripheral samples were 34.4%-60.5% and 35.5%-64.0%, respectively. Platelet counts were, conversely, lower in peripheral samples compared to cord samples. The average difference was -22.5 × 10^3^/mm^3^ with an average cord Plt of 264.8 × 10^3^/mm^3^ versus 242.3 × 10^3^/mm^3^ peripherally. The ranges of Plt values from cord versus peripheral samples were 118-400 × 10^3^/mm^3^ and 110-384 × 10^3^/mm^3^, respectively.

**Table 2:** Cord Versus Peripheral Complete Blood Count (CBC) Results WBC: white blood cell; HgB: hemoglobin; Hct: hematocrit; Plt: platelets; SD: standard deviation

	Cord (mean ± SD)	Peripheral (mean ± SD)	Correlation (R)	p-value
WBC (×10^3^/mm^3^)	14.1 ± 6.1	15.6 ± 6.2	0.826	<0.001
HgB (g/dL)	15.8 ± 1.9	16.8 ± 2	0.608	<0.001
Hct (%)	47 ± 5.6	48.3 ± 5.7	0.653	0.008
Plt (×10^3^/mm^3^)	264.8 ± 56.4	242.3 ± 56.4	0.684	<0.001

WBC from peripheral samples was higher. The average difference was 1.5 × 10^3^/mm^3^ with an average cord WBC of 14.1 × 10^3^/mm^3^ versus 15.6 × 10^3^/mm^3^ peripherally. The ranges of WBC values from cord versus peripheral samples were 3.2-33 × 10^3^/mm^3^ and 2.5-37.5 × 10^3^/mm^3^, respectively. As the correlations were between 0.6 and 0.9, the data suggest only moderate predictability between cord and peripheral blood samples for WBC. Data from the MD from cord and peripheral samples were reviewed (Table 3). The average immature/total neutrophil count ratio was different with cord versus peripheral samples of 0.06 and 0.09, respectively.

**Table 3:** Cord Versus Peripheral Manual Differential (MD) Results I:T ratio: immature/total neutrophil count ratio; SD: standard deviation

	Cord (mean ± SD)	Peripheral (mean ± SD)	Correlation (R)	p-value
Neutrophils (%)	41.7 ± 13.7	46 ± 12.3	0.430	0.007
Bands (%)	2.5 ± 3.7	4.3 ± 5.6	0.531	0.001
Lymphocytes (%)	36.8 ± 13.8	33.5 ± 13	0.479	0.027
Monocytes (%)	11.5 ± 5.1	9.6 ± 3.9	0.278	0.002
Eosinophils (%)	2.1 ± 2.2	1.9 ± 2.3	0.310	0.510
Metamyelocytes/myelocytes (%)	1 ± 1.5	1.1 ± 1.6	0.429	0.373
I:T ratio	0.06 ± 0.09	0.09 ± 0.10	0.411	0.038

Another aspect of our study was further analysis and comparison of the samples drawn from infants with and without documented DCC (Table 4). We found evidence of increased peripheral HgB and Hct values in those infants who received any DCC with nearly a threefold (0.6-1.7 g/dL) increase in the difference between peripheral to cord HgB with a history of DCC, whereas WBC and Plt counts were not different. The impact of time between cord and peripheral samples was also evaluated (Table 5). There were no differences among WBC or HgB/Hct when comparing samples obtained more than or less than one hour apart. The Plt count dropped when samples were obtained greater than one hour after birth. Most peripheral samples were obtained less than four hours after birth, and the timing of the blood draw was consistently documented by nursing annotation.

**Table 4:** Effects of Delayed Cord Clamping (DCC) on Complete Blood Count (CBC) Differences WBC: white blood cell; HgB: hemoglobin; Hct: hematocrit; Plt: platelets; SEM: standard error of the mean

	DCC (mean ± SEM)	No DCC (mean ± SEM)	p-value
Peripheral - cord WBC (×10^3^/mm^3^)	1.5 ± 0.4	1.5 ± 0.4	0.052
Peripheral - cord HgB (g/dL)	1.7 ± 0.2	0.6 ± 0.2	0.010
Peripheral - cord Hct (%)	3.2 ± 0.5	0.1 ± 0.5	0.009
Peripheral - cord Plt (×10^3^/mm^3^)	-18.2 ± 5.0	-21.7 ± 5.0	0.537

**Table 5:** Effects of Timing on Complete Blood Count (CBC) Differences WBC: white blood cell; HgB: hemoglobin; Hct: hematocrit; Plt: platelets; SEM: standard error of the mean

	More than one hour between samples (mean ± SEM)	Less than one hour between samples (mean ± SEM)	p-value
Peripheral - cord WBC (×10^3^/mm^3^)	0.6 ± 0.4	1.5 ± 0.4	0.222
Peripheral - cord HgB (g/dL)	0.9 ± 0.2	1.0 ± 0.2	0.958
Peripheral - cord Hct (%)	0.4 ± 0.5	1.7 ± 0.5	0.181
Peripheral - cord Plt (×10^3^/mm^3^)	-39.9 ± 4.9	-6 ± 4.9	<0.001

## Discussion

Complete blood counts for infants admitted to the NICU or newborn nursery are routine [1-3]. We demonstrated the feasibility of utilizing umbilical cord blood after placental delivery for this test.

Our results show that WBC, HgB, and Hct values obtained from cord blood trend lower than those obtained from peripheral samples as opposed to Plt counts that trend higher in cord blood samples. Cord and peripheral blood values follow a predictable pattern, remain within accepted normal ranges, and can be used clinically [16]. Our data suggest that the well-established concept of postnatal fluid shifts and fluid losses contributing to the increasing HgB, Hct, and WBC values may be occurring immediately after birth [21]. However, the lower Plt counts seen in peripheral samples cannot be explained by postnatal fluid shifts. We speculate that the transitioning fetal to postnatal circulation (i.e., vasospasm of the umbilical vessels, ductus arteriosus, and ductus venosus) may reflect a mild progressive, consumptive process and thus a slight downtrend in total Plt count within the first few hours after birth. Although the cause of the lower Plt count is not understood at this time, our study corroborates with previous studies comparing umbilical and peripheral CBC indices [4,8,10]. We identified only one study that demonstrated that cord and peripheral CBCs were neither statistically nor clinically different from each other and also concluded that both samples fall within clinically acceptable ranges [18].

Delayed cord clamping increases the HgB concentration in neonates [7,14]. Our data are the first to show more disparate cord versus peripheral values with DCC compared with immediate cord clamping. However, regardless of DCC, none of our peripheral samples demonstrated a Hct greater than 65%, which would prompt repeat sampling to rule out polycythemia per our current practice [22]. All cord and peripheral samples had Hct greater than 30% and less than 65%. Our sample set did not include any infants who had perinatal cord accidents or hemorrhagic events such as avulsion, prolapse, or abruption, which would underreport neonatal anemia if cord samples were used prior to equilibration from the acute blood loss. In these situations, a peripherally drawn sample would be recommended.

We believe that anecdotal concerns have been raised regarding the potential effects of Wharton’s jelly interfering with proper heme analyzer function. There is no literature reporting this interaction, and our laboratory did not experience analyzer malfunction. Our collection protocol advises against collecting blood samples through Wharton’s jelly deposits. Our experience supports other published reports for cord blood testing on various hematology analyzers that also do not cite or mention Wharton’s jelly interference [20,23].

Another aspect of our data that bears further evaluation is the potential cost and time savings of drawing blood from the cord for admission laboratory tests compared to peripheral samples. As our delivery team already draws blood routinely for type and screen and cord blood gases, there would be no increase in venipuncture supplies or staff support to add a CBC compared with a separate phlebotomy procedure at a later time. As cord blood is also validated for microbiology cultures at our hospital, the potential exists for asymptomatic newborns to be spared any direct venipuncture procedures as part of an infectious screen. This could not only benefit the hospital and infant-maternal dyads from a financial standpoint but also improve dyad bonding and breastfeeding success by limiting the separation of the infant for laboratory tests during the first few hours of life.

The strengths of our study include a diverse sample size consisting of various ages and ethnic backgrounds. Additionally, we report data on DCC’s effects on CBC differences via comparison to their respective cord samples and also report observations from the time between cord and peripheral sample collections. With proper education and phlebotomy technique, there was not a single instance of contamination with Wharton’s jelly affecting the heme analyzer’s functional operation. One limitation was that DCC became more widespread over the enrollment period, such that only 24% of infants in the earlier cohort underwent DCC compared with 54% in the later cohort. However, it did allow us to analyze CBC differences between DCC and immediate cord clamping. As 12% of peripherally drawn sample sources were not documented in the medical record, we are not able to definitively comment on the inherent variability between free-flowing and capillary samples. Capillary samples tend to be more hemoconcentrated compared to free-flowing venous or arterial samples; therefore, the degree of difference between a capillary sample and a cord sample could be significant compared to that of a venous or arterial sample. Our hospital practice favors the arterial sampling technique that makes peripherally collected samples more homogeneous. We also recognize that the benefits of CBC cord blood sampling may be nominal for late preterm and term infants apart from avoiding a phlebotomy procedure. Finally, the cord blood source (umbilical vein versus artery) was not differentiated as we, like other clinical practices, concluded that there are no clinical CBC differences between the two sources [24].

## Conclusions

Cord blood is an acceptable source for CBC sampling in newborn infants at our institution. By utilizing cord blood, infants may avoid undergoing painful phlebotomy procedures. Cord blood CBC values are predictably different from peripheral blood but remain within acceptable ranges. Our data also suggest that recent clinical practice changes, such as DCC, do not alter the utility of cord blood for clinical decision-making. Our findings contribute to an increasing body of normative cord blood values.
